# Dinámica de circulación de virus sincicial respiratorio y su relación con la temperatura ambiental. Serie de 25 años en Buenos Aires

**DOI:** 10.31053/1853.0605.v80.n3.40438

**Published:** 2023-09-29

**Authors:** Fernando Torres, Rosana Abrutzky, Paula Domínguez, Javier Potasznik, Gabriela Sanluis Fenelli, María José Rial, María Fabiana Ossorio, Fernando Ferrero

**Affiliations:** 1 Hospital General de Niños Pedro de Elizalde.; 2 Universidad de Buenos Aires. Facultad de Ciencias Sociales

**Keywords:** virus sincicial respiratorio humano, infecciones del tracto respiratorio, cambio climático, calentamiento global, human respiratory syncytial virus, respiratory tract infections, climate change, global warmingy, vírus sincicial respiratório humano, infecções respiratórias, mudança climática, aquecimento global

## Abstract

**Introducción:**

El cambio climático global podría alterar la circulación del virus sincicial respiratorio (VSR).

**Objetivo:**

Evaluar modificaciones en la circulación de VSR en los últimos 25 años y su correlación con la temperatura ambiente.

**Métodos:**

Estudio transversal, utilizando registros de VSR y temperatura de la Ciudad de Buenos Aires (1995-2019). Para cada año, describimos inicio, fin y duración de la temporada de VSR y evaluamos su correlación con la temperatura media anual.

**Resultados:**

Se identificaron 10183 infecciones por VSR. La duración de la temporada disminuyó significativamente (1995: 29 semanas vs. 2019: 18 semanas; R: 0.6 (p< 0,001)), debido a una finalización más precoz (1995: semana 45 vs. 2019: semana 34; 0,6 (p<0,001)). No se observó correlación entre temperatura media anual y duración, comienzo ni finalización de la temporada de VSR.

**Conclusión:**

En los últimos 25 años, la duración de la temporada de VSR se acortó significativamente, sin correlación con la temperatura.

## Abstract (en)

**Background:**

Global climate change could modify the seasonality of the Respiratory Syncytial Virus (RSV).

**Objective:**

To evaluate changes in RSV seasonality in the last 25 years and their correlation with the mean annual temperature.

**Methods:**

Cross-sectional study, based on RSV and temperature data from the City of Buenos Aires (1995-2019). For each year, we describe the beginning, end, and duration of the RSV season and the correlation with the mean annual temperature.

**Results:**

We identified 10,183 RSV infections. The duration of the RSV season decreased significantly (1995: 29 weeks vs. 2019: 18 weeks; R: 0.6 (p< 0.001)), due to an earlier ending (1995: week 45 vs. 2019: week 34; 0.6 (p<0.001)). No correlation was observed between mean annual temperature and the duration, start, or end of the RSV season.

**Conclusion:**

In the last 25 years, the duration of the RSV season has been significantly shortened due to an earlier ending, without correlation with temperature.

## Resumo

**Introdução:**

A mudança climática global pode alterar a circulação do vírus sincicial respiratório (VSR).

**Objetivo:**

Avaliar as modificações na circulação do VSR nos últimos 25 anos e sua correlação com a temperatura ambiente.

**Métodos:**

Estudo transversal, utilizando registros de VSR e temperatura da Cidade de Buenos Aires (1995-2019). Para cada ano, descrevemos o início, fim e duração da temporada do VSR e avaliamos sua correlação com a temperatura média anual.

**Resultados:**

Foram identificadas 10.183 infecções por VSR. A duração da temporada diminuiu significativamente (1995: 29 semanas vs. 2019: 18 semanas; R: 0,6 (p<0,001)), devido a um final mais precoce (1995: semana 45 vs. 2019: semana 34; 0,6 (p<0,001)). Não foi observada correlação entre a temperatura média anual e a duração, início ou finalização da temporada do VSR.

**Conclusão:**

Nos últimos 25 anos, a duração da temporada do VSR foi significativamente reduzida, sem correlação com a temperatura.

**CONCEPTOS CLAVE**

Que se sabe sobre el tema.

El virus sincicial respiratorio representa una alta carga de enfermedad en la infancia, con un patrón epidémico en países de clima templado. El cambio climático global produce un constante aumento de las temperaturas medias anuales

Que aporta este trabajo.

Este trabajo describe de manera objetiva la circulación del virus sincicial respiratorio mostrando una finalización más precoz de la duración de las temporadas a lo largo de una serie de 25 años previos a la pandemia y que podría estar asociada a un mayor número de casos en el inicio de cada temporada.La calidad de vida es un factor importante en el rendimiento académico de los alumnos universitarios.

Conocer la calidad de vida es la base para diseñar políticas universitarias acordes a la realidad del alumnado.

**Divulgación**

El cambio climático global podría modificar los patrones epidemiológicos de algunas infecciones en niños. Este trabajo presenta las modificaciones observadas en la circulación del virus sincicial respiratorio (inicio, finalización, y duración de la temporada) en una serie de 25 años sobre pacientes internados en un hospital pediátrico, y la correlación de la misma con las variaciones observadas en las temperaturas medias anuales en el mismo periodo. Además, explora la relación entre la cantidad de casos en las primeras semanas de la temporada y la duración de la misma.

## Introducción

El virus sincicial respiratorio (VSR) continúa siendo el principal patógeno respiratorio en la infancia, con una importante carga de enfermedad en este período de la vida ^1^ . En países de clima templado su circulación muestra un patrón epidémico en los meses más fríos ^2^ .

El cambio climático global podría afectar este patrón de circulación, llevando a temporadas más cortas ^3^ .

Por otro lado, ya sea por causas estrictamente epidémicas o por fenómenos sociales asociados, la pandemia por COVID 19 impactó enormemente en la circulación de los virus respiratorios habituales (incluyendo el VSR), cambios que aún persisten, hasta cierto punto, incluso 3 años después del inicio de este fenómeno ^4^ .

Para poder valorar adecuadamente en los próximos años la circulación de VSR en el contexto post pandémico y con un cambio climático que se acelera ^5^ , es fundamental contar con datos sólidos del período anterior.

Nosotros ya estudiamos la circulación del VSR en la Ciudad de Buenos Aires entre los años 1995 y 2014 ^6^ , encontrando que la temporada se ha ido acortando a lo largo de ese período. En este estudio buscamos extender ese análisis a un intervalo más extenso, que incluya el período pre-pandémico inmediato, de manera de contar con una sólida base para analizar el comportamiento del virus una vez finalizada la pandemia.

Nuestro objetivo fue evaluar si la temporada de VSR se modificó en un período de 25 años y si esa modificación mostró correlación con cambios en las temperaturas medias anuales. También, buscamos explorar algunas características de la dinámica de circulación del VSR en cada temporada.

## Métodos

Estudio transversal incluyendo datos de un período de 25 años previos previo al inicio de la pandemia por COVID 19 (1995-2019). Se utilizaron registros de microbiología (VSR) de un hospital pediátrico de la Ciudad de Buenos Aires y datos oficiales de temperatura de la Ciudad de Buenos Aires aportados por el Servicio Meteorológico Nacional (SMN). Se recabó el número de casos de VSR por semana epidemiológica y temperaturas medias anuales (media, mínima y máxima).

Análisis: Para cada año, describimos el inicio, el fin y la duración de la temporada de VSR, calculados de acuerdo a lo consignado en un estudio anterior (6). Por otro lado, describimos las temperaturas medias, medias mínima y media máxima anuales de cada año. Las variaciones de cada una de las mencionadas variables a lo largo del período en estudio fueron evaluadas por regresión lineal simple.

También se compararon los promedios de semanas de duración, inicio y fin entre las décadas inicial (1995-2004) y final (2010-2019) del período de estudio con la prueba t de Student.

Además, se evaluó la correlación entre las temperaturas medias (media, mínima y máxima) anuales y el inicio y fin de la temporada de VSR (número de semana epidemiológica) y su duración (en semanas), mediante la correlación de Pearson (coeficiente de determinación R).

Finalmente, se exploró si la cantidad de casos de VSR al inicio de la temporada incide en la duración de la misma (análisis de curva ROC, chi cuadrado y regresión).

Consideraciones éticas: Los datos de temperatura fueron provistos por el SMN. Los referidos a VSR fueron provistos por el servicio de microbiología del HGNPE, absolutamente disociados de cualquier dato filiatorio. Se solicitó y obtuvo autorización del Comité de Ética en Investigación. El estudio se inscribió en el Registro Público de Investigaciones del Gobierno de la Ciudad de Buenos Aires (7618/2022).

## Resultados

En el período estudiado se identificaron 10183 infecciones por VSR. Analizamos las temperaturas medias anuales y el comienzo, la finalización y la duración de la temporada de VSR (Tabla 1).

**Tabla N°1:** Temporada de circulación de virus sincicial respiratorio (VSR) y temperatura anual promedio en la Ciudad de Buenos Aires

	Temporada VSR				Temperatura media anual		
	Inicio	Finalización	Duración		Media	Mínima	Máxima
	(SE)	(SE)	(semanas)		(°C)	(°C)	(°C)
1995	17	45	29		17,81	13,47	22,98
1996	20	47	28		18,31	13,71	23,38
1997	13	39	27		18,52	14,29	23,3
1998	17	46	30		17,84	13,56	23,36
1999	11	40	35		17,74	13,43	22,41
2000	12	39	29		17,71	13,62	22,31
2001	16	47	24		18,38	14,37	22,85
2002	16	40	32		17,99	13,78	22,73
2003	18	46	24		17,69	13,28	22,72
2004	17	36	30		18,06	13,83	23,08
2005	18	38	21		17,93	13,46	23,01
2006	17	39	21		18,16	13,86	23,24
2007	17	33	17		17,43	12,89	22,45
2008	18	36	19		18,52	14,25	23,81
2009	17	39	23		18,19	13,78	23,45
2010	14	28	15		18,24	13,77	23,09
2011	14	30	17		17,95	13,65	22,92
2012	17	32	16		18,29	13,95	23,45
2013	18	36	19		18,06	13,66	23,38
2014	17	33	17		18,32	14,01	23,26
2015	16	36	21		18,55	14,25	23,47
2016	17	30	14		17,75	13,65	22,54
2017	17	33	17		18,83	14,63	23,68
2018	18	35	18		18,59	14,5	23,38
2019	17	34	18		18,66	14,18	23,25

SE: semana epidemiológica

El inicio de la temporada de VSR fue en la semana 17 tanto en 1995 como en 2019 (la semana de inicio más precoz fue la 11 y la más tardía, la 20; mediana= 17; R: 0,1; p= 0,2). La finalización de la temporada de VSR fue en la semana 45 en 1995 y en la 34 en 2019 (la semana de finalización más precoz fue la 28 y la más tardía, la 47; mediana= 26; R: 0,6; p <0,001). La duración de la temporada de VSR fue de 29 semanas en 1995 y 18 en 2019 (la menor duración fue de 14 semanas y la mayor, de 35; mediana= 21; R: 0,6; p 0,001) (Figura 1).

**Figura N° 1:**
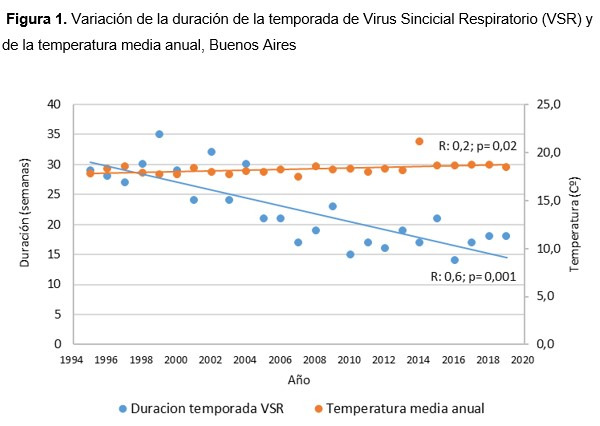
Variación de la duración de la temporada de Virus Sincicial Respiratorio (VSR) y de la temperatura media anual, Buenos Aires.

Al comparar la primera década del período (1995-2004) con la última (2010-2019), observamos menor duración de la temporada de VSR (28,8 ± 3,3 vs. 17,2 ± 1,9 semanas; p <0,001), con finalización más precoz (semana 42,5 ± 4,1 vs. semana 32,7 ± 2,7; p <0,001), y sin diferencias en el inicio de la misma (semana 15,7 ± 2,8 vs. semana 16,5 ± 1,4; p= 0,4) (Tabla 2).

**Tabla N° 2:** Modificaciones en la temporada de virus sincicial respiratorio (VSR) y en la temperatura media anual, Ciudad de Buenos Aires

		Década inicial	Década final	pǂ
(1995-2004)	(2010-2019)
Temporada VSR	Duración*	28,8 ± 3,3	17,2 ± 1,9	<0,001
	Inicial**	15,7 ± 2,8	16,5 ± 1,4	0,4
	Final**	42,5 ± 4,1	32,7 ± 2,7	<0,001
Temperatura media anual (°C)ǂǂ	Media	18,0 ± 0,3	18,2 ± 0,3	0,04
	Máxima	22,9 ± 0,3	23,2 ± 0,3	0,05
	Mínima	13,7 ± 0,3	14,0 ± 0,3	0,08

*en semanas **número de semana epidemiológica

^ǂ^ Prueba de T para muestras independientes

^ǂǂ^ Media ± DS

La temperatura media anual fue 17,4ºC en 1995 y 18,4ºC en 2019, con un aumento promedio de 0,04ºC por año (R: 0,2; p= 0,02) (Figura 1). Dicho aumento no se verificó en la temperatura media máxima anual (R: 0,3; p= 0,1) pero si en la temperatura media mínima anual (R: 0,4; p= 0,04).

Al evaluar la correlación entre las temperaturas medias (media, mínima y máxima) anuales y el inicio, el fin y la duración de la temporada de VSR, solo se encontró una débil correlación entre el inicio de la temporada y la temperatura media máxima anual (R:0,2; p: 0,01) (Tabla 3).

**Tabla N° 3:** Correlación entre la circulación de virus sincicial respiratorio (VSR) y la temperatura media anual. Ciudad de Buenos Aires

		Temperatura media anual		
		Media	Mínima	Máxima
		r (R)	r (R)	r (R)
Temporada VSR	Duración	-0,3 (0,09)	-0,2 (0,04)	-0,3 (0,09)
	Inicio	0,2 (0,04)	0,1 (0,01)	-0,5 (0,2) *
	Final	-0,2 (0,04)	-0,1 (0,01)	-0,1 (0,01)

*p<0,05

También se evaluó el impacto del número de casos en el inicio de la temporada en su duración. Para ello se determinó el punto de corte del número de casos en las primeras 8 semanas que permitiera predecir si la temporada duraría por debajo o por encima del promedio de duración de las 25 temporadas (22,4 semanas). La curva ROC (auc= 0,8 IC95% 0,6-0,9) identificó a menos de 100 casos en las primeras 8 semanas como el valor que mejor puede identificar una duración de la temporada superior al promedio (7/8 en los de <100 casos vs. 4/17 en los de >100 de casos; OR= 22,7 IC95% 2,1-244,8 p=0,04).

Completando este análisis, también verificamos un aumento del número total de casos (R:0,1 p=0,05), número de casos en las primeras 8 semanas (R:0,4 p=0,001) y proporción de casos en las primeras 8 semanas (R:0,3 p=0,03) a lo largo de la serie.

## Discusión

En este estudio confirmamos que la duración de la temporada de VSR se acortó significativamente en la Ciudad de Buenos Aires en los últimos 25 años. Este cambio se verificó a expensas de una finalización más precoz, manteniendo el inicio en la misma época del año.

Existe evidencia sobre el incremento de las temperaturas tanto a nivel global ^7^ como local ^8^ relacionados con el cambio climático global. Nosotros pudimos verificar un incremento en la temperatura media anual en el período estudiado, pero no pudimos demostrar una correlación significativa entre la duración de la temporada de VSR y el aumento de la temperatura, como evidenció Donaldson en Inglaterra ^3^ .

Sin embargo, la tendencia es manifiesta, al punto que hubiera sido suficiente un incremento de 0,1 °C en las temperaturas medias de los últimos 5 años del período estudiado para evidenciar una correlación inversa significativa entre ambas variables (temperatura y duración de la temporada de VSR). Sin embargo, la alteración que sufrió la circulación del VSR durante la pandemia no nos permitirá verificarlo en el corto plazo, al interrumpir nuestra serie de manera impactante ^4^ .

Por otro lado, es razonable hipotetizar sobre qué otros fenómenos, que no sean los cambios en la temperatura, pueden explicar la disminución en la duración de las temporadas de VSR que encontramos.

En este sentido, es posible que, a lo largo de los 25 años estudiados hayan ocurrido fenómenos sociales que impacten en la circulación viral. Dado que la mayoría de los pacientes en los que se identifica VSR son lactantes ^9^ , es posible que la interacción más precoz entre pares de ese rango etario favorezca un contagio más extensivo. En los últimos 25 años ha aumentado la proporción de niños que concurren a centros de cuidado diurno ^10^ . También en los últimos 25 años, el aumento de la pobreza en el área de estudio ^11^ es posible que haya incrementado el hacinamiento y la proporción de población que concurre a comedores comunitarios ^12^ y, consecuentemente, la interacción social.

La circulación de los diferentes virus ha sido estudiada en muchas oportunidades. Una de las premisas más establecidas es que la disminución en el número de susceptibles disminuye y aún anula la circulación viral ^13^ . En nuestro estudio encontramos que un mayor número de casos en el inicio de la temporada predice una temporada de menor duración. Esto indicaría que, una temporada con mayor número de casos en su inicio agotaría más rápidamente el número de susceptibles y limitaría la circulación viral. Esta observación se refuerza con la correlación que observamos entre cada vez mayor proporción de casos en el inicio de la temporada y temporada más cortas.

Nuestro estudio aporta información sobre la duración de la temporada del virus que más frecuentemente ocasiona hospitalizaciones en pediatría. Estos datos podrían ayudar a ajustar la respuesta del sistema de salud frente a cada temporada invernal en término de previsión de recursos humanos y materiales ^14^ .

También brinda información útil para, eventualmente, adecuar los programas de prevención de la infección por VSR con la administración de anticuerpos monoclonales (palivizumab y nirsevimab) ^15^ .

Este estudio tiene la fortaleza de basarse en datos de una sola institución que utilizó el mismo procedimiento diagnóstico durante todo el período y los mismos criterios para indicar la pesquisa microbiológica. Sin embargo, esto mismo se transforma en una limitación para extrapolar los resultados a otros escenarios.

## Conclusión

En los últimos 25 años, la duración de la temporada de VSR se acortó significativamente, sin mostrar, aún, correlación con la temperatura ambiente. Las temporadas de VSR con mayor número de casos en su inicio tienden a ser más cortas.

## References

[1] Li Y, Wang X, Blau DM, Caballero MT, Feikin DR, Gill CJ, Madhi SA, Omer SB, Simões EAF, Campbell H, Pariente AB, Bardach D, Bassat Q, Casalegno JS, Chakhunashvili G, Crawford N, Danilenko D, Do LAH, Echavarria M, Gentile A, Gordon A, Heikkinen T, Huang QS, Jullien S, Krishnan A, Lopez EL, Markić J, Mira-Iglesias A, Moore HC, Moyes J, Mwananyanda L, Nokes DJ, Noordeen F, Obodai E, Palani N, Romero C, Salimi V, Satav A, Seo E, Shchomak Z, Singleton R, Stolyarov K, Stoszek SK, von Gottberg A, Wurzel D, Yoshida LM, Yung CF, Zar HJ, Nair H, Respiratory Virus Global Epidemiology Network, RESCEU investigators (2022). Global, regional, and national disease burden estimates of acute lower respiratory infections due to respiratory syncytial virus in children younger than 5 years in 2019: a systematic analysis. Lancet.

[2] Obando-Pacheco P, Justicia-Grande AJ, Rivero-Calle I, Rodríguez-Tenreiro C, Sly P, Ramilo O, Mejías A, Baraldi E, Papadopoulos NG, Nair H, Nunes MC, Kragten-Tabatabaie L, Heikkinen T, Greenough A, Stein RT, Manzoni P, Bont L, Martinón-Torres F (2018). Respiratory Syncytial Virus Seasonality: A Global Overview. J Infect Dis.

[3] Donaldson GC (2006). Climate change and the end of the respiratory syncytial virus season. Clin Infect Dis.

[4] Ferrero F, Ossorio MF, Rial MJ (2022). The return of RSV during the COVID-19 pandemic. Pediatr Pulmonol.

[5] Tollefson J (2022). Climate change is hitting the planet faster than scientists originally thought. Nature.

[6] Ferrero F, Torres F, Abrutzky R, Ossorio MF, Marcos A, Ferrario C, Rial MJ (2016). Seasonality of respiratory syncytial virus in Buenos Aires. Relationship with global climate change. Arch Argent Pediatr.

[7] Lindsey R, Dahlman L Climate Change: Global Temperature.

[8] Barros VR, Boninsegna JA, Camilloni IA, Chidiak M, Magrín GO, Rusticucci M (2015). Climate change in Argentina: Trends, projections, impacts and adaptation. WIREs Clim Change.

[9] Fujiogi M, Goto T, Yasunaga H, Fujishiro J, Mansbach JM, Camargo CA Jr, Hasegawa K (2019). Trends in Bronchiolitis Hospitalizations in the United States: 2000-2016. Pediatrics.

[10] Schuez-Havupalo L, Toivonen L, Karppinen S, Kaljonen A, Peltola V (2017). Daycare attendance and respiratory tract infections: a prospective birth cohort study. BMJ Open.

[11] Gasparini L, Tornarolli L, Gluzmann P (2019). El desafío de la pobreza en Argentina. Diagnóstico y perspectivas.

[12] (2022). Millions in Argentina relying on food banks and soup kitchens as prices surge. Buenos Aires Times.

[13] Burrell CJ, Howard CR, Murphy FA (2017). Epidemiology of Viral Infections. Fenner and White's Medical Virology.

[14] Paes BA, Craig C, Pigott W, Latchman A (2013). Seasonal respiratory syncytial virus prophylaxis based on predetermined dates versus regional surveillance data. Pediatr Infect Dis J.

[15] Panozzo CA, Stockman LJ, Curns AT, Anderson LJ (2010). Use of respiratory syncytial virus surveillance data to optimize the timing of immunoprophylaxis. Pediatrics.

